# Tell me about your hay fever: a qualitative investigation of allergic rhinitis management from the perspective of the patient

**DOI:** 10.1038/s41533-018-0071-0

**Published:** 2018-01-23

**Authors:** Biljana Cvetkovski, Vicky Kritikos, Kwok Yan, Sinthia Bosnic-Anticevich

**Affiliations:** 1 University of Sydney, Sydney, Australia; 2 Woolcock Institute, University of Sydney, Sydney, Australia; 3 Royal Prince Alfred Hospital, Sydney, Australia; 4 Sydney Local Health District, Sydney, Australia

## Abstract

Allergic rhinitis (AR) is sub-optimally managed in the community and is responsible for a significant health and economic burden. Uncontrolled AR increases the risk of poorly controlled asthma and presents an increased susceptibility to thunderstorm asthma. With the availability of treatments over-the-counter, bypassing the health care professional (HCP), the role of the patient is paramount. Research on the role of the patient in AR management in the current environment is limited. This study aims to explore the patient perspective of AR management and understand why it is sub-optimally managed in the community. Patient perspectives of AR management were explored utilizing a qualitative, phenomenological approach. Adults with AR were included in the study and interviewed. Transcripts were analyzed for recurrent themes and emergent concepts. Forty-seven participants with AR were interviewed about their experiences. Patient reports of delayed diagnosis, treatment fatigue and confidence in the ability to manage their AR themselves, heavily influenced their management preferences. Patients also described barriers associated with AR management including financial expense as well as being mistaken for having an infectious disease. Patients described examples of the impact on their quality of life caused by their AR, yet they strongly believed they could manage it themselves. This belief that AR is a condition that should be entirely self-managed, contributes to its burden. It amplifies patients’ separation from HCPs and having access to guidelines aimed at optimizing their AR control.

## Allergic rhinitis: Reaching beyond over-the-counter medications

Healthcare professionals must engage fully with allergic rhinitis patients to optimize treatment and avoid issues triggered by constant self-care. Allergic rhinitis, or hay fever, can impact hugely on individuals’ health but remains poorly managed, partly because of the multiple medications available that allow people to bypass doctors and treat themselves. Biljana Cvetkovski at the University of Sydney, Australia, and co-workers, interviewed 47 adults with hay fever to analyze perceptions of the condition and its management. Many patients reported disenchantment with professional guidance for hay fever, often citing delayed diagnosis and endless tests leading to long-term ‘treatment fatigue’ and a loss of confidence in healthcare services. Patients often prefer self-treatment with limited medical help, repeatedly switching between medications and opting for ‘quick fixes’ rather than treating the chronic condition, with sometimes serious consequences.

## Introduction

In 2014-2015, allergic rhinitis (AR) affected 4.5 million i.e., 19% of Australians, becoming the most prevalent respiratory condition in the mid-teenage years; peaking in working aged adults.^1^ Its prevalence and poor control is of worldwide concern.^2–7^ The burden of this highly prevalent respiratory condition, while often trivialized, is staggering, with uncontrolled AR impacting on the quality of life (QOL), work productivity, school performance, social interactions driving ability and sleep^8–10^ of those who suffer from the condition.

In Australia, the burden of AR has recently been highlighted as a consequence of a fatal “Thunderstorm Asthma” event.^11,12^ During this event, an unprecedented, unexpected demand was made on health care facilities by people in respiratory distress, 90% of which had a history of AR.^13,14^ Questions arose about how such a fatal event could have been avoided, given the availability of comprehensive, evidence-based guidelines both for the treatment of AR and asthma.^15–19^

AR management guidelines present a step-wise strategy, which couple allergen (trigger) avoidance, medication management (including over-the-counter [OTC] and prescription medicines), and immunotherapy in specific, severe cases of AR. However, despite this multidimensional approach, the reality is that allergen avoidance is not effective for most patients and immunotherapy is limited to only a very select group of patients, thereby the mainstay treatment of AR relies on medication management.^20^ Therefore, in exploring the burden of AR, attention must be drawn to the medication management of AR by health care providers and the subsequent medication self-management practices of individuals with AR.^21–25^ Of particular interest is the latter.

It is well recognized that patient self-management plays a major role in the successful management of chronic illnesses.^26–28^ While this has been explored in the related field of asthma, in which patient medication self-management has been shown to play a significant role in overall treatment success;^29^ our understanding of medication self management in AR is limited. While large-scale studies in Europe and the Americas have provided us with detailed information about patient medication preferences along with the co-existence of bothersome symptoms and loss of productivity,^3,22–25,30,31^ they fail to explain why patients chose to self-medicate in the way they do, despite often living with sub-optimally managed AR.

The aim of this study is to gain an in-depth understanding of the experiences, beliefs and reasons behind the medication self-management practices of patients with AR.

## Results

Fifty-seven people contacted the research team expressing interest in participating in the study. Of these, forty-seven people with AR from New South Wales, Australia, were interviewed for this study, with the remainder not being able to be contacted by the research team following initial expression of interest. The 47 participants, were aged from 18 to 86 years of age with a median age of 39 and 28 were female. Eight participants were from rural communities in NSW and 39 from the Sydney metropolitan area. Interview duration ranged from 9 to 67 min with an average duration of 23 min. Four interviews were conducted face-to-face at the Woolcock Institute and the remainder were conducted over the telephone. Participants who chose to participate over the telephone were sent participant information and consent forms via email or post based on their preference. The majority of recruited participants were on the Woolcock’s volunteers database and had responded to the letters of invitation or had heard of the study through snowballing.

Data saturation was obtained following the forty-second interview after which no new information was described by the subsequent participants. However all forty-seven volunteers were interviewed to ensure saturation.

Seven themes were identified that best represented the patient’s perspective of AR management. The semi-structured interview guide appears in Table 1. This already appears in the methods Data Collection Section.

**Table 1:** Semi-structured interview guide

Primary question	Secondary questions	Clarification
Tell me about your experiences with allergic rhinitis/hay fever (symptoms, challenges, satisfaction)	How long have you been experiencing these symptoms?	What times of day/year do you experience worsening symptoms?
	What treatments do you currently or have previously tried for your symptoms?	What do you find makes them better or worse?
	What prompted you to try these treatments?	
	How would you describe the impact of these symptoms on your daily activities?	

### Delayed diagnosis and misdiagnosis

A large proportion of participants reported a challenge associated with getting a correct diagnosis. In most of these cases, they had experienced symptoms from childhood and reported symptoms for many years. They were assumed to have a cold or flu and expressed relief at the point when it had been diagnosed as AR. A huge burden being lifted as a result of knowing what their symptoms were caused by, rather than relief of symptoms. They reported seeing multiple HCPs before a suitable treatment was found.

Why do I always have a cold? P13

There was also a subset of participants who had identified as having developed AR in adulthood. They reported a delay in diagnosis, they did not expect to develop AR later in life, often they had never experienced any symptoms earlier in life and although less common, some participants reported having being treated for asthma and only later being diagnosed as having AR instead.

I’m 52 and I didn’t get asthma until I was in my 40 s and then a few years ago I had what I felt was a cough that just kept going on and on. The doctor said no, it was hay fever. So it was allergic rhinitis. P10

### Treatment fatigue

The delay in diagnosis of AR, especially in childhood often meant participants had been exposed to a gamut of tests and treatments prior to and following their diagnosis of AR. They described a childhood filled with many investigations, medications, doctor’s appointments and alternative treatment options.

My parents did everything under the sun. They’ve gone everywhere. P31

As a result of this, they reported a sense of treatment fatigue, i.e., a sense of having explored all available options with regards to treating their AR and having exhausted any desire to pursue further investigations or treatment options. They had concluded that there was nothing available to eliminate their symptoms. They had also developed skepticism about products that claimed to be “new” and would eliminate AR. Participants no longer believed that there were any options that they had not explored and that all new products were similar to old products, just in new packaging or marketed differently. They felt they could no longer generate energy to explore “new” treatments as they assumed they would be disappointed with the efficacy as they had been in the past with other products with similar claims. Consequently, these participants no longer enquired about treatments with HCPs nor did they do their own research about AR. They stated that the only thing that sparked their interest to reconsider their AR management were media reports about a “breakthrough” in AR treatment.

I keep my ears open for breakthroughs, but there aren’t any. P1

There’s just nothing I can do to relieve it and the other thing too is I’ve given up buying drugs over the counter because I know they don’t work. P10

### Confidence in their ability to manage AR themselves

Participants reported confidence in their ability to manage their AR independently. After years of trying different medications and consulting HCPs, patients felt that they had learnt enough and tried enough treatments to determine for themselves what was best. This was especially the case when considering symptom relief, identifying risk factors and preferences with regards to mode of treatment delivery. With regards to symptom relief, the participants expressed a high tolerance of their symptoms and were satisfied with a small reduction in symptom severity.

As soon as I take them all the symptoms dissipate. They won’t certainly go away. But they certainly dissipate. P30

So Zyrtec, Beconase, the sprays. Like the nasal sprays and the tablets, whatever brand really. Claratyne, I’ve tried all of them. P57

The chronic nature of AR allowed them to recognize patterns of symptoms, identify triggers and develop avoidance strategies. Patients felt that HCP appointments were a significant impingement on their own time and that their own efforts in managing their AR were a more efficient use of their time. Using their own skills to manage their AR let them accommodate their treatment preferences best, both with respect to method of delivery and expectation regarding symptom relief. Some participants prepared for a predicted worsening of symptoms due to an upcoming exposure to triggers by taking antihistamines preventatively, based on their own experimentation and belief for what is best for them.

I usually get itchy eyes when the pollen count is really high and also when it’s dusty and I sneeze a lot as well. P18

That is more of a preventative measure - I stock up on it 2 weeks in advance - which minimises the effect of the symptoms. Heading up to spring and so the body, I believe, builds up a sort of immunisation or builds up a resistance to the hay fever. It allows me to deal with it on a - in a much better fashion. P31

### Management preferences

There were three predominant sentiments that were reported by participants when describing their preferences for managing their AR, all of which related to medication use and selection. Firstly, a preference not to medicate at all with the exception of a nasal saline wash or mist. Secondly, those who reluctantly medicate, when they felt the symptoms were otherwise intolerable. Thirdly, those who want to take any form of treatment in order to be symptom free.

The first group that had a preference not to medicate, represented people who did not believe that medication worked and those that had beliefs against medication. A subset of participants felt that they had tried all available AR treatments and nothing cured or completely eliminated their symptoms, therefore taking symptom-relieving medicines was a waste of time and an unnecessary risk of experiencing adverse effects. Other participants who wished not to medicate, said that they ideologically preferred not to use medicines for their AR.

So I don’t really like sticking chemicals up my nose. P25

I want someone to tell me that just having a carrot a day or something gross like a zucchini will fix everything. I’ll be like ‘ok, I’ll do it if I really have to’. P17

I don’t particularly like taking medication. P19

The second group medicated when they perceived it necessary, sharing some of the sentiments of participants who preferred not to medicate at all. They too felt that no treatment completely eliminated their symptoms, hence did not sense a purpose in using regular medicines and experiencing the expense and commitment associated with it. They preferred not to have to use any medicines, but in some circumstances perceived their symptoms to be intolerable and unacceptably impeding on their productivity or quality of life. In such circumstances, this cohort of participants had a preference for using non-sedating oral antihistamines for their ease of use, availability, onset of action and perceived effectiveness. The majority of participants had at some point used an intranasal corticosteroid [INCS], however very few were still using it. The main reason for discontinuation was the perception that it did not work, even though they had used it for weeks or months at a time. Some participants reported they had undergone immunotherapy at some stage in their lives. For some, this meant a significant reduction in their symptoms and symptoms thereafter were manageable with the occasional use of oral antihistamines. For others, it cemented the ideology that nothing would cure their AR and these participants were not motivated to try any further treatments.

I wait for the symptoms to occur because I’m very anti-drug and the symptoms are so severe to the point where I actually can’t work, because it is like a head cold. P30

I have to take something otherwise I am a mess. Only when it gets really really bad. A couple of times a week. P56

I’d use them just while there is a lot of flowers and dust in the air, the change of season in springtime and that sort of thing. If it started coming on then I’d use it for a week or something and see how I went without it. P50

The final group, which medicated to be symptom free, used more medicines more frequently than the other two groups. AR presented a significant burden to them and their quality of life that they were willing to take regular medicines preventatively to try and control their symptoms. This cohort of participants reported an exploration of alternate medicines in order to relieve the symptoms of AR. They explored alternate therapies in circumstances where traditional medicines had not met their needs. These participants were most likely to have undergone immunotherapy.

[What prompted you to see a naturopath in the first place?]

Just desperation I think. P21

I’d have to rely on them daily. To be able to cope, yeah…to work I’d have to rely on them - even the eye sprays - to get me through the day because it gets really bad, really irritating. They [symptoms] just reduce for a few hours. P39

An individuals’ approach/attitude to taking AR medicine was not influenced by the severity of symptoms or the impact on quality of life. Regardless of the participant’s severity or medication preferences, there was an overwhelming preference for use of oral medicines due to ease of administration and use, portability, palatability, perceived effectiveness and onset of action (compared with INCS). Participants reported that when they were using a medicine, which they perceived as adequately relieving their symptoms, they would not explore alternative options. There was however a belief among the majority of participants that they would develop a tolerance to oral antihistamine medicines if they repeatedly used the same product. They believed that this could be avoided if they occasionally were to switch brands.

I alternated between them, but I found the Claratyne was the most effective, so I ended up just buying Claratyne from then on. P56

No it[non-sedating antihistamine] seems to be working well, cost effective, I don’t get any side effects from it, but yeah. P44

Because [antihistamines] are smaller and I can swallow them without water. P13

I didn’t find them [INCS] that effective. I still use them if I feel like I have to.

But I just don’t find them that effective. So I don’t really rely on them to do

what they need to do. P57

Some, participants had consulted an ear, nose, and throat surgeon. Where additional nasal issues were identified and ear-marked as potentially benefitting from surgical intervention (e.g., deviated septum), there were mixed responses in the uptake. There was almost an equal distribution between people who were willing to explore surgery as an option versus those who preferred to avoid it. Some thought a one-off intervention was worth considering where others felt that the risk and significance of surgery substantially outweighed any potential benefit. Some participants reported a significant improvement in the ability to breathe after they had surgery, only then realizing that they had accepted a poor standard of breathing in the past.

### Financial expense

Participants reported that in some circumstances their choices regarding AR management were based on cost. Cost arose as an issue when describing treatment selections, visits to a doctor and procedures to formally diagnose AR. The perception of financial burden of AR treatments was evaluated based on the person’s perception of its monetary value versus perceived effectiveness. In some instances further investigation, such as a skin prick test, was deemed expensive and were not perceived as necessary. Participants felt they had identified their triggers through observation of pattern and exposure history. Similarly, the financial expense of medication was questioned based on how effective it was perceived to be and the difference it made to the person’s quality of life.

[Preference for medication], yeah by price basically. P34

A lot of it depends on how expensive they are as well. P41

That’s always been a goal of mine to be able to have the pre-test to find out exactly what the triggers are. But I pretty much know what they are-know not specifically, but I know what they are. Pollens and dust pretty much. It doesn’t really bother me, so I haven’t done it and it’s expensive. P50

It worked quite good[INCS] but at the time I didn’t have the money to buy it because it was so expensive. P25

I had to use it-to do one spray of the steroid one and another spray of the other one otherwise it wouldn’t work. Until eventually the steroid did take over but it didn’t happen straight away. It was really annoying because I’d spent all this money on the spray but I had to use the other one. P25

### Unwanted attention

There was a strong sentiment among the participants that they did not want to be labeled as having an illness. They referred to it as nuisance or an irritation, rather than a chronic respiratory disease. For some, this meant that they deliberately did not discuss their AR with anyone and internalized their management entirely. They felt that discussing it with others would not achieve anything except bring them more unwarranted attention.

The whole idea is, in front of people I want to just come across as unaffected. P47

Participants reported that the symptoms of AR brought unwarranted attention, particularly sneezing and nasal congestion. They felt that there is a stigma associated with sneezing and nasal congestion due to others misinterpreting the symptoms as belonging to a contagious cold or flu. Participants reported having to constantly explain to people that they have AR and are not infectious and do not need to be isolated or kept away from school or the workplace. The symptoms of AR also caused them to feel embarrassed among their colleagues and peers.

There’s a big stigma and focus on people sneezing…so I often say to people I don’t have a cold, it’s hay fever. P14

It’s embarrassing you know, having these symptoms. P14

People think I’ve got a cold. PH

We’ve got a bridge club and they always say, ‘if you’ve got a cold, stay home, we don’t want you here’. I’ve had to tell people, ‘well it isn’t a cold that I have. I’ve just got it all the time’. P7

### Impact on wellbeing

A theme that repeatedly displayed variation among participants was the perception of the impact AR on their life. Responses ranged from AR being regarded as an “inconvenience” to something that if persistent, can make them feel depressed. Participants who felt AR was a nuisance, regardless of its severity, did so because they compared it to having a life-threatening condition. Ultimately, they felt that AR did not warrant more attention than they had given it because it would not end their life.

You get not depressed but kind of like depressed I suppose the word is. It’s very debilitating. You can’t believe it but it’s true. P33

It is an annoyance, but it’s not a lethal, life threatening disease or anything. It’s not life crippling. It’s just inconvenient, really. P56

No, no that bad [not symptoms everyday]. I’d neck myself if it was every day. P2

Many identified the impact of AR on daily activities extended beyond themselves and their immediate family.

I have a young family now so sleep is a little more precious to me [than it used to be, hence have sought referral to a specialist]. P8

People with AR were more likely to notice the impact of AR on their life when it interfered with their workplace productivity. Treatment options were explored and determined based on how AR affected their ability to perform at work. Participants became tolerant to a certain impact on their quality of life brought upon by AR. This tolerance would be challenged and further treatment would be sought when the impact on quality of life was heightened, sometimes due to factors unrelated to AR, (e.g., poor sleep quality associated with AR was no longer tolerated when sleep quality was also challenged with the arrival of a newborn baby).

I’m tired and it’s hard to concentrate and feel unwell. P14

To go to work, I’d have to rely on them [antihistamines],even the eye sprays, to get me through the day because it gets really bad, really irritating. P39

I can’t work, now I’m allergic to rats… I couldn’t continue with research in that area. A career working with animals would have been out for sure. P14

## Discussion

This research provides important fresh insights to the reasons why people with AR sub-optimally manage their condition and goes to explain what has led to patients choosing to carry the burden of AR management themselves. This study of patient’s perspective has identified that patient’s choice to self-manage their AR is linked to sub-optimal experiences with HCPs and confidence in their own abilities to treat their AR with OTC medicines. AR guidelines are developed for use by HCPs^32^ and by self-managing their AR in isolation, patients are moving further away from the reach of these guidelines and into unguided territory, compounding the burden it has on the community.

In conducting this research, one striking point was noted; when patients consider the management of their AR, there is a dominant focus on medication management. While self-management is multifaceted^33^ and includes elements of trigger avoidance and monitoring in addition to medication management, the patient’s perspective of management and their role in it is focused on pharmaceuticals and the options they have. There are several aspects that impact on this, as highlighted in the following discussion.

The delayed diagnosis/misdiagnosis of AR plays a formative role in the patient’s sub-optimal management. Disenchantment with HCPs’ ability to identify their problem steers them on the path of self-management, in a sense, towards a “self-help” approach. This goes to set up a vicious cycle, and increased burden of AR, as delayed diagnosis results in a delay in seeking medical advice until symptoms are intolerable,^3^ a finding similar to that experienced by patients with chronic rhinosinusitus.^34^

Treatment fatigue (defined as a decreased desire to explore further treatment based on exhaustion from pursuing previous unsuccessful pathways) was synonymous with and often a consequence of delayed diagnosis. Treatment fatigue caused patients to be reluctant to explore further therapies or consult HCPs. While treatment fatigue is often associated with poor adherence,^35^ treatment fatigue in AR seems to be less about adherence and more about disillusionment with multiple failed attempts to control their AR with the treatments available. Treatment fatigue in AR also expands to the patient’s rigmarole of health care consultations and investigative procedures, without AR symptom resolution. To overcome treatment fatigue, patients must be offered support and counseling regarding their AR as has been shown for other chronic respiratory disorders such as asthma.^36^

Patients believe that AR is a condition that they should be self-managing. This is influenced by the availability of medicines over the counter (OTC). As patients try different medicines, they develop confidence in their ability to do so, continuing the cycle of self-management and treatment trials. Being able to choose their medicines OTC^37^ facilitates them in accessing their preferred oral, quick relief, occasional-use medicine and avoid the disliked intranasal sprays.^23,38^ The preference for quick relief occasional-use medicine is evidence of their perception of AR as an acute condition, rather than a chronic disease.

This research highlights that it is not just the availability of OTC medicines that drives the patient to self-manage their AR. These patients reported that they know what works best for them and suggested that their HCPs have not entirely understood their goals for their AR. Understanding of patients’ treatment aims and goals has been shown to improve their AR management^39–41^ and should be taken into account when developing their management plan.

In considering these findings it is important to consider possible solutions. Possible solutions should include simple pathways for recognition and diagnosis of AR in primary care,^42^ and the development of tools and support mechanism for patients through therapeutic partnerships with HCPs.^43^ Of critical importance, is that tools available to patients should be dynamic, accommodate for changing AR severities and provide guidance in instances of a “flare up” as seen in written asthma action plans.^33^ The MACVIA-Allergy Diary App^44^ is able to provide the patient with some guidance on self-managing their AR but further research needs to be conducted to investigate how HCPs can best collaborate with patients to optimize its use.

This study was potentially limited by the sample, which is likely to under-represent patients with mild AR. While a majority of participants reported being diagnosed with AR, the inability to confirm the patient’s diagnosis of AR is a potential limitation. It should be noted however that the demographics of the study population were consistent with that reported previously for the Asia-Pacific region.^38^

In conclusion, our findings strongly demonstrate that the patient’s experience with AR is unique, varied and very much a condition in which patients self-manage with medications that are easily and abundantly available to them. AR management is influenced by patients’ experiences prior to receiving a diagnosis following which their knowledge and beliefs about AR and its treatments are formed. These results highlight the critical need to increase the dialog between HCPs and their patients regarding AR, improve the understanding of the significance of AR and optimize its management and for HCPs to acknowledge patients’ expectations and beliefs with regards to treatment. HCPs must engage patients, embrace AR self-management and work collaboratively with patients to optimize their AR management.

## Methods

This study took the form of a qualitative exploration of patients’ perspectives of AR management and was conducted as a part of a larger study exploring AR management in the community.

### Study design

A phenomenological approach was utilized to explore the phenomenon of AR. Participants were asked to describe their experiences with AR and given “free range” to voice what AR meant to them.

### Participant recruitment

*Inclusion criteria:* People aged 18 years or older, who identified themselves as having AR and were able to speak English, were eligible for inclusion in the study.

*Recruitment:* Advertisements for the study were placed on the website and Facebook page of the Woolcock Institute of Medical Research, Sydney, Australia. Letters of invitation were sent to individuals with AR on a volunteers’ database at the Woolcock Institute (a database created for the purpose of connecting patients with respiratory and sleep conditions, who have responded to advertisement fro study participation). People with AR, who expressed interest in participating, were required to contact the research team to give consent and make an appointment to be interviewed. Snowballing was welcomed and participants were permitted to alert others with AR about the study. Participants did not receive any reward or remuneration for participating in the study.

### Data collection

Participants were given the option of being interviewed in person at the Woolcock Institute or over the telephone. Interviews were conducted by one researcher (BC). They were audio recorded on a digital audio recording device, transcribed verbatim and stored electronically. Participants were informed that their responses were completely de-identified and would not be traced back to them as individuals in any circumstances. Written participant consent was obtained prior to the commencement of the study.

*Sample size:* Phenomenological research requires interviews to be conducted with 5–25 people.^45^ Participant recruitment continued until data saturation was achieved with consideration of the phenomenological research sampling frame guidelines.^46^

*Semi-structured interview guide:* The semi-structured interview guide, based on empirical data and a phenomenological framework^32,47,48^ covered a range of topics/facets of AR management (including pharmaceutical management, non-pharmaceutical management, perceived impact on quality of life, barriers and challenges associated with AR and recognition of patterns of AR flare-ups) and served to guide the qualitative framework of the research.^45^

The interview commenced with open ended questioning in which participants voiced their initial thoughts or their most pertinent perceived issues. Secondary and clarifying questions were asked to encourage further thoughts and experiences to be articulated.

The specific questions included in the interview guide are displayed in Table 1. If during the interviews, information arose that had not been considered previously, BC would make a note to explore this point in subsequent interviews.

### Data analysis

*Qualitative data analysis and representation:* In phenomenological data analysis, “significant statements” that represent how the participant experienced the phenomenon are identified as “codes”. These codes are then categorized into themes that are used to write a description of the experiences of the participants with the phenomenon being researched.^45^

A mixed deductive—inductive approach was utilized in the qualitative analysis of the interview transcripts. The mixed analysis approach sought to identify themes arising from the identification of codes in AR literature (deductive) as well as identification of codes from the analytical reading of the data (inductive). The deductive analysis identified the participants’ responses to themes that were known to be important with regards to AR management in the literature. Such codes included treatment preferences, symptoms, investigations and HCP consultations. These themes were often prompted with the interview guide if not immediately described by the participant. Inductive analysis of the transcripts included the identification of codes that were presented organically through the interview process but were not necessarily explored by the interview guide.^45^

The researchers (BC, SBA, and VK) met weekly to review a summary of the interviews conducted during the week based on notes taken by BC, while conducting the interviews. Data saturation was identified following reports of no new perspectives being provided by participants with regards to their AR management.

The initial process of analysis involved a detailed description of what BC and SBA independently observed in the transcripts and categorizing those observations into codes. Some of these codes represented deductive analysis e.g. descriptions of medicines used while others were inductive and were observed without pre-determined identification of the code, e.g. being mistaken for having a cold. Following the independent categorization conducted by BC and SBA, meetings were held with VK and KY to discuss the codes and the transcripts, look for common elements and categorize them into agreed themes. The breadth and scale of interviews meant that there were many individual and unique perspectives of AR management described by the participants, where the volume is prohibitive of all being reported in this paper. BC and SBA found that participants gave very detailed accounts of the pharmaceutical regimens they use for managing their AR. Codes describing participants pharmaceutical management of AR were categorized into themes by BC and SBA, describing their preferences and the reasons behind them, rather than detailed descriptions of the regimen. In addition to the descriptions of pharmaceutical regimens BC and SBA identified common themes overarching many of the transcripts. These themes were chosen to be reported in this manuscript based on what BC, SBA, VK, and KY perceived to be most important to the participants during their interviews. Further results not reported here will be reported at later stage in subsequent manuscripts.

ATLAS.ti was used to facilitate the qualitative analysis by providing an organizational structure to link the codes, themes and their supporting quotes among the interview transcripts.^49^

Once analysis was complete, the participants were not given the opportunity to provide feedback on the findings but were able to request a summary of the results.

This study was approved by the University of Sydney Human Ethics Committee. Methods were performed in accordance with COREQ regulations and guidelines.^50^
